# Exploring the Utility of Circulating Endothelial Cell-Derived Extracellular Vesicles as Markers of Health and Damage of Vasal Endothelium in Systemic Sclerosis Patients Treated with Iloprost

**DOI:** 10.3390/biomedicines12020295

**Published:** 2024-01-27

**Authors:** Giuseppe Argentino, Bianca Olivieri, Alessandro Barbieri, Ruggero Beri, Caterina Bason, Simonetta Friso, Elisa Tinazzi

**Affiliations:** 1 Department of Medicine, University of Verona, 37134 Verona, Italy; biancaolivieri92@gmail.com (B.O.); ruggero.beri@univr.it (R.B.); caterina.bason@univr.it (C.B.); simonetta.friso@univr.it (S.F.); elisa.tinazzi@aovr.veneto.it (E.T.); 2 Department of Laboratory Medicine, Boston Children’s Hospital, Harvard Medical School, Boston, MA 02115, USA; alessandro.barbieri@childrens.harvard.edu

**Keywords:** extracellular vesicles, microparticles, endothelial cells, systemic sclerosis, apoptosis, annexin V, CD144, CD146

## Abstract

Endothelial cell-derived extracellular vesicles (eEVs) are released from endothelial cells, signifying endothelial integrity. Systemic Sclerosis (SSc) is a rare disease causing skin and organ fibrosis with early vascular damage. Iloprost, an SSc treatment, might affect eEV release, showing long-term benefits. We aimed to study eEVs in SSc, potentially serving as disease markers and linked to Iloprost’s impact on organ involvement. We included 54 SSc patients and 15 healthy donors. Using flow cytometry on platelet-poor plasma (PPP) with specific antibodies (CD144, CD146, AnnexinV), we detected endothelial extracellular vesicles. Results showed fewer eEVs from apoptotic or normal cells in SSc patients than healthy controls. Specifically, patients with diffuse cutaneous SSc and lung issues had reduced eEVs from apoptotic endothelial cells (CD146+ AnnV+). No notable differences were seen in CD144 endothelial markers between patients and controls. After 1-day Iloprost infusion, there was an increase in eEVs, but not after 5 days. These findings suggest circulating eEVs reflect endothelial health/damage, crucial in early SSc stages. A 1-day Iloprost infusion seems effective in repairing endothelial damage, critical in scleroderma vasculopathy. Differences in marker outcomes may relate to CD146’s surface expression and CD144’s junctional location in endothelial cells.

## 1. Introduction

Systemic Sclerosis (SSc) is a rare multisystemic disease of unclear etiology, characterized by altered immune response, widespread vascular dysfunction, and progressive fibrosis affecting both the skin and internal organs. It presents a range of symptoms, varying in severity and progression. Common symptoms include skin tightening, Raynaud’s phenomenon, joint pain, and fatigue. Internal organs can also be affected, leading to complications in the digestive system, heart, lungs (interstitial lung disease and pulmonary arterial hypertension), and kidneys.

According to the extent of skin involvement, two different subsets of the disease are traditionally recognized: limited cutaneous (lcSSc) and diffuse cutaneous (dcSSc) Systemic Sclerosis. LcSSc typically presents with skin thickening confined to the hands, arms, and face. It progresses more slowly and is less likely to involve internal organs severely. Raynaud’s phenomenon is often an early sign in lcSSc. On the other hand, dcSSc involves more extensive skin thickening, affecting larger areas of the body, including the trunk. This subtype tends to progress more rapidly and is more likely to involve internal organs, particularly the kidneys, heart, and lungs. Patients with dcSSc may experience a quicker onset of symptoms and more severe complications compared to those with lcSSc. The pathogenesis of SSc involves three central mechanisms: endothelial cell damage, immune system dysfunction, and fibroblast cells activation [1,2,3,4].

Vascular damage is considered an early event in the disease course, resulting from increased endothelial cell apoptosis and impaired reparative pathways. This ultimately leads to a loss of capillaries, localized hypoxia, and subsequent fibrotic responses [5,6,7]. Auto-antibodies such as anti-Scl70, anti-RNA polymerase III, and anticentromere contribute to disease subset classification. However, current biomarkers lack sensitivity and specificity for monitoring vascular damage progression [8,9,10]. Vasculopathy in SSc results from an abnormal imbalance between defective vasculogenesis and angiogenesis. The precise mechanisms behind vascular changes are largely unknown. Histologically, SSc is characterized by a gradual loss of small vessels, capillary dilation, and arteriole/small artery stenosis. Vascular damage progresses in SSc patients, leading to faulty vascular remodeling and angiogenesis. Pro-angiogenic factors like VEGF, ET-1, and adhesion molecules are overexpressed, while anti-angiogenic factors such as angiostatin, CXCL-4, and IL-4 are elevated in SSc patients [11].

Within this context, circulating extracellular vesicles (ectosomes: microparticles/microvesicles; EVs) [12,13] shed by endothelial cells have recently emerged as potential indicators of endothelial integrity [14,15] and have also been studied in other diseases as potential biomarkers [16,17]. These EVs range from 100 nm to 1000 nm in diameter, and are released by various cell types and platelets through membrane blebbing under both pathological and physiological conditions [18]. Comprising a dual phospholipid layer, EVs carry membrane proteins reflecting their cellular origin and can be promptly identified using cell surface marker staining [19].

Among the endothelial cell-derived EV (eEV) markers, CD144 or Vascular Endothelial (VE)-cadherin [20] and CD146, also known as Melanoma Cell Adhesion Molecule (MCAM) [21], are used for their specificity. CD144 is a glycoprotein crucial for cell-cell adhesion, playing a vital role in maintaining the structural integrity of adherent junctions between endothelial cells. It is primarily involved in reinforcing the endothelial barrier [20] and has a role in various biological processes, such as trans-endothelial migration of leukocytes [22], angiogenesis, and vascular development during the embryonic period [20]. It is expressed by endothelial cells, circulating endothelial progenitor cells, and CD34+ hematopoietic stem cells [23]. CD146, on the other hand, is part of the immunoglobulin superfamily and functions in the intercellular adhesion between endothelial cells. Unlike CD144, which is confined to adherent junctions, CD146 has a more diverse localization within the plasma membrane of endothelial cells [21]. It also acts as a receptor for the extracellular matrix component, laminin-α4, and it is primarily expressed in various endothelial cells, including those in junctions, circulating mature cells, and to a lesser extent, late endothelial precursors. Additionally, it is found in other vessel wall cells like smooth muscle cells and pericytes, as well as a limited number of activated T and B lymphocytes, mesenchymal stem cells, and melanoma cells [24]. While other markers like CD31 and CD51 have been used to identify the endothelial origin of EVs, they are not exclusive to endothelial cells and appear on other cell types like platelets and macrophages [23]. Additionally, Annexin V (Ann V) is often used to distinguish EVs derived from apoptotic cells by binding to exposed phosphatidylserine on the outer membrane, thus serving as an apoptosis marker [25].

The potential of eEVs as markers for endothelial dysfunction in SSc remains ambiguous, possibly due to various confounding factors. These include the timing of eEV detection relative to the disease stage and treatment interventions, especially those known for their protective effects on endothelial function [26]. Iloprost, a prostacyclin analog, is commonly used to treat SSc patients, particularly in cases featuring ischemic ulcers or pulmonary hypertension [27]. Iloprost activates adenylate cyclase, leading to the production of cyclic AMP (cAMP) through the stimulation of prostaglandin I2 (PGI2) receptors found on smooth muscle cells and endothelial cells. The activation of these receptors results in the suppression of smooth muscle constriction. In addition to its vasodilatory properties, Iloprost inhibits platelet activation and aggregation and appears to enhance the turnover of endothelial progenitor cells, thereby potentially mitigating endothelial damage. It also supports the formation and strengthening of endothelial adherens junctions, decreasing the permeability of the endothelial cell monolayer. Notably, adherens junctions play a crucial role in enhancing Nitric Oxide (NO) signaling, inhibiting cell apoptosis, and reducing inflammation [28]. This function may also influence the release of eEVs. However, to date, no study has evaluated extracellular endothelial vesicles and their changes in peripheral blood concerning Iloprost infusion.

Our study aims to explore the utility of eEVs as disease markers in SSc and to assess any correlations with organ involvement and Iloprost infusion. Additionally, we will explore which surface marker is the most reliable for evaluating endothelial cell-derived extracellular vesicles.

## 2. Patients and Methods

### 2.1. Patients

We enrolled 54 patients diagnosed with SSc who were referred to the Autoimmune Diseases Unit, Department of Medicine, University Hospital of Verona, along with 15 healthy donors.

All patients met the classification criteria for SSc established by the American College of Rheumatology (ACR) and the European League Against Rheumatism (EULAR) in 2013 [29]. The classification of patients into diffuse cutaneous and limited cutaneous forms of the disease was based on LeRoy’s criteria [30], which were later reviewed in 2001 to incorporate the Early Scleroderma diagnosis [31]. These criteria are based on clinical features, such as Raynaud’s phenomenon, SSc-related autoantibodies (anti-centromere, anti-topoisomerase I, anti-fibrillarin, anti-PM-Scl, anti-RNA polymerase I or III) and capillaroscopy results (dilatation and/or avascular areas).

Among the enrolled patients, 21 had dcSSc, and 33 had lcSSc. Various organ involvements were observed in the patients: 7 had active digital ulcers, 39 had gastrointestinal involvement (such as hiatal hernia, gastroesophageal reflux, esophagus-cardial atony, or chronic gastritis detected through esophagogastroduodenoscopy, esophageal manometry, or barium meal), 35 showed pulmonary involvement, including 28 with interstitial lung disease (ILD) (including 10 subjects with interstitial disease detected by high-resolution lung CT scan (HRCT) but without clinical symptoms), 2 patients had pulmonary arterial hypertension (PAH) confirmed by echocardiography and right heart catheterization, and 5 patients had both interstitial disease and PAH. Additionally, 24 subjects presented secondary Sjogren’s syndrome. Table 1 provides a summary of the clinical data.

All patients had been on a stable treatment regimen for at least six months, including endothelin-receptor antagonists, calcium channel blockers, and mycophenolate mofetil (see Table 1), as well as cyclic infusion of Iloprost (details provided in the next section, “Iloprost treatment”).

The study included 15 healthy controls who were matched with the patients in terms of sex and age. The control group consisted of 14 females and 1 male, with an average age of 51 ± 8 years (range: 39–63). None of the controls had a history or signs of autoimmune, infectious, cardiovascular, or metabolic diseases. At the time of sample collection, none of the controls were taking any medications.

The study protocol adhered to the Helsinki Declaration of 1975 (revised in 2000), and it was approved by the local Ethical Committee (protocol number 1538, version number 3). All patients and controls provided informed consent to participate in this study.

### 2.2. Iloprost Treatment

All patients received intravenous Iloprost infusion (Endoprost 0.05 mg/0.5 mL) at a variable dose ranging from 0.5 to 2 ng/kg/min, depending on individual tolerance. The patients participating in this study were divided into two groups based on the schedule of Iloprost infusion. One subgroup received a single-day monthly infusion, while the other subgroup received 5-day daily infusion every three months. Twenty-five patients underwent monthly Iloprost infusion, with eighteen having limited SSc and seven having diffuse SSc. Fourteen patients followed the 5-day infusion schedule, with seven having the limited form of SSc and seven having the diffuse form. The choice between these two regimens was influenced by individual patient factors such as tolerance to the treatment, as well as logistical considerations like patient accessibility to the treatment center. Circulating extracellular vesicles were collected and analyzed before and after the Iloprost infusion for both groups of patients.

### 2.3. Blood Preparation and Flow Cytometric Detection of eEVs

Venous peripheral blood samples were processed within one hour of collection in tubes containing sodium citrate. Platelet-poor plasma (PPP) was obtained through two sequential centrifugations: the first at 1800× *g* for 10 min at 25 °C and the second at 3000× *g* for 10 min at 25 °C [32].

Extracellular vesicles in the PPP were directly analyzed using flow cytometry (FACSanto II, BD Bioscience, San Jose, CA, USA) with monoclonal antibodies [32,33,34]: anti-CD144−PE (phycoerythrin) or anti-CD146−PE-CY7 (phycoerythrin-cyanin7) (BD Bioscience) to identify their endothelial origin, and anti-Annexin V-APC (Allophycocyanin; AnnV) (BD Bioscience) to identify extracellular vesicles derived from apoptotic cells. For each analyzed sample, two tubes were prepared: the first tube contained a combination of anti-CD144−PE and anti-AnnV-APC, while the second tube contained a combination of anti-CD146−PE-CY7 and anti-AnnV-APC. A morphological gate was designed using calibration beads (Flow Cytometry Size Calibration Kit; Life Technologies, Carlsbad, CA, USA) to identify extracellular vesicles with a diameter of less than 1 μm. Data analysis was performed using FlowJo 10 software (Treestor, Ashland, OR, USA). The extracellular vesicles included in the gate were further subdivided based on the expression of CD144 or CD146 and AnnV on their surface: (I) extracellular vesicles derived from normal endothelial cells (CD144+/146+AnnV−), (II) extracellular vesicles derived from apoptotic endothelial cells (CD144+/146+AnnV+), (III) extracellular vesicles derived from non-endothelial cells, both viable (CD144−/146−AnnV−) and (IV) apoptotic (CD144−/146−AnnV+) (Figure S1).

### 2.4. Statistical Analysis

The data obtained from flow cytometry were analyzed using GraphPad Prism 10 software (GraphPad Software Inc., La Jolla, CA, USA) for statistical analysis. All data are presented as mean ± standard deviation (SD). The statistical correlations between the data were assessed using the Mann–Whitney test. Statistical significance was considered when the *p*-value was <0.05, indicating significant differences between the groups.

## 3. Results

### 3.1. Difference in Endothelial Extracellular Vesicle Percentages between Patients and Controls

**Figure 1:**
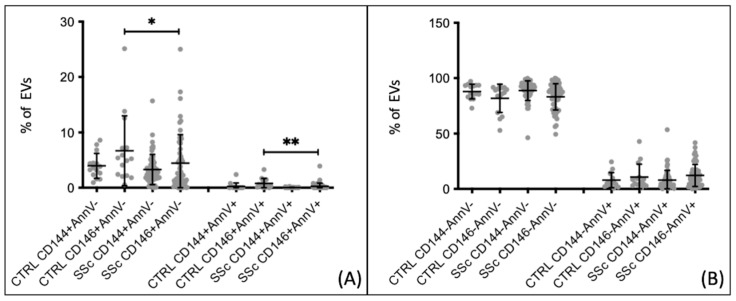
Endothelial and non-endothelial extracellular vesicles percentages comparison between SSc patients and healthy controls. (**A**) eEV CD146+ populations from apoptotic and non-apoptotic cells are lower in SSc patients than in CTRLs. (**B**) EVs, both CD144− and CD146−, do not show significant differences. CTRL *n* = 15, SSc *n* = 54. * *p* < 0.05, ** *p* < 0.005.

**Figure 2:**
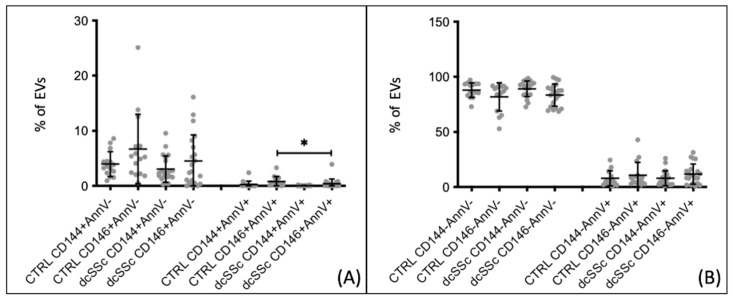
Endothelial and non-endothelial extracellular vesicles percentages comparison between dcSSc patients and healthy controls. (**A**) eEV CD146+ populations from apoptotic are lower in dcSSc patients than in CTRLs. (**B**) EVs, both CD144 and CD146, do not show significant differences. CTRL *n* = 15, dcSSc *n* = 21. * *p* < 0.05.

**Figure 3:**
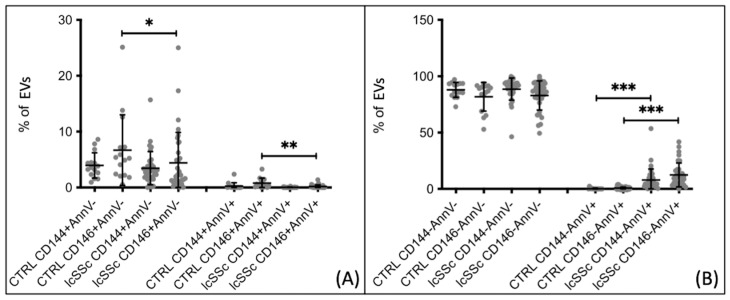
Endothelial and non-endothelial extracellular vesicle percentages comparison between lcSSc patients and healthy controls. (**A**) eEV CD146+ populations from apoptotic and non-apoptotic cells are lower in lcSSc patients than in CTRLs. (**B**) EV CD144− and CD146− populations from apoptotic cells are higher in lcSSc patients than in CTRLs. CTRL *n* = 15, lcSSc *n* = 33. * *p* < 0.05, ** *p* < 0.005, *** *p* < 0.0005.

Endothelial extracellular vesicles CD146+, originating from either live and/or activated cells (AnnV−) or apoptotic cells (AnnV+), were found to be lower in patients with SSc as compared to healthy controls. These results were obtained from samples collected under basal conditions at least 1 month after the last Iloprost infusion. Patients with Systemic Sclerosis exhibited a lower percentage of CD146+ AnnV− extracellular vesicles compared to healthy controls (4.448 ± 5.155 vs. 6.691 ± 6.332, *p* = 0.029). The same pattern was observed for CD146+ AnnV+ extracellular vesicles (0.263 ± 0.568 vs. 0.796 ± 0.898, *p* = 0.0015) (Figure 1A). However, these differences were not confirmed when analyzing endothelial extracellular vesicles using the CD144 marker. No statistically significant differences were found in the percentages of non-endothelial extracellular vesicles (CD144−/146−), both apoptotic (AnnV+) and derived from live/activated cells (AnnV-), between SSc patients and healthy controls (Figure 1B). Subsequently, we compared the two subgroups of SSc patients with healthy controls. A decrease in apoptotic endothelial extracellular vesicle percentages (CD146+ AnnV+) was observed in patients with dcSSc compared to healthy controls (0.376 ± 0.850 vs. 0.796 ± 0.898, *p* = 0.0111) (Figure 2A). These findings were not observed when using the CD144 marker. Additionally, there were no differences in the percentages of non-endothelial extracellular vesicles (CD144−/146−) between patients and controls (Figure 2B). When comparing extracellular vesicle percentages between lcSSc patients and healthy controls, significant differences were found for both endothelial and non-endothelial extracellular vesicles. Patients exhibited a lower percentage of CD146+ endothelial extracellular vesicles derived from live/activated cells compared to controls (4.407 ± 5.462 vs. 6.691 ± 6.332, *p* = 0.0281). Moreover, patients displayed a decreased percentage of CD146+ endothelial extracellular vesicles derived from apoptotic cells compared to controls (0.193 ± 0.277 vs. 0.796 ± 0.899, *p* = 0.0018). These differences were not observed for the CD144 marker (Figure 3A). In terms of non-endothelial extracellular vesicles, significant differences were detected for the subgroup derived from apoptotic cells. In patients with lcSSc, the CD144− AnnV+ extracellular vesicles were higher than those in healthy controls (7.909 ± 9.890 vs. 0.244 ± 0.626, *p* < 0.0001). This pattern was also observed for the CD146 marker, with increased CD146− AnnV+ extracellular vesicles compared to healthy controls (12.430 ± 10.590 vs. 0.696 ± 0.898, *p* < 0.0001) (Figure 3B). Thus, the most notable aspect of this initial analysis is the decrease in both live and apoptotic endothelial-derived extracellular vesicles in SSc patients.

### 3.2. Difference in Endothelial and Apoptotic Extracellular Vesicle Percentages between Patients

The correlation between the percentages of extracellular vesicles and the capillaroscopic pattern was evaluated, and no significant differences were found in the percentages of extracellular vesicles among patients with different capillaroscopic patterns (Figure S2).

There were also no significant differences in the percentages of extracellular vesicles between patients with active ulcers and those without (Figure S3) or among patients with or without pulmonary involvement (Figure S4).

**Figure 4:**
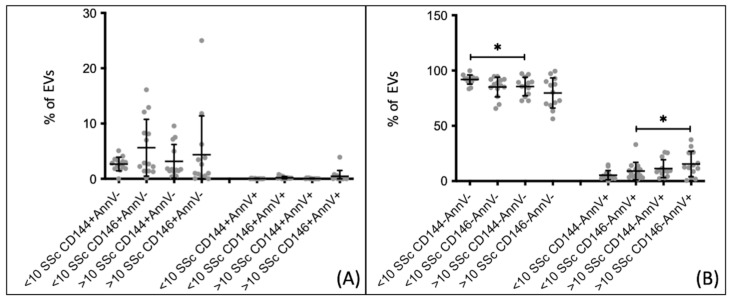
Endothelial and non-endothelial extracellular vesicles comparison between SSc patients with different duration of the disease. (**A**) eEVs, both CD144+ and CD146+, do not show significant differences. (**B**) EVs CD144− from non-apoptotic cells are lower in patients with more than 10 years of the disease (>10), while EVs CD146− from apoptotic cells are lower in patients with less than 10 years of the disease (<10). <10 SSc *n* = 14; >10 SSc *n* = 13. * *p* < 0.05.

The correlation between the percentages of extracellular vesicles and the duration of the disease was also examined. Since SSc is characterized by vascular remodeling that leads to vessel obliteration over time, the patients were divided into two groups based on the duration of the disease, using a 10-year cut-off point as an arbitrary division. This information was available for 27 out of the 54 patients included in the study. The percentages of endothelial extracellular vesicles, both apoptotic and derived from live/activated cells, did not show statistically significant differences in relation to the duration of the disease (Figure 4A). However, significant differences were observed for non-endothelial CD144− extracellular vesicles between the group of patients with a disease duration of less than 10 years and those with a longer duration. The percentages of CD144− AnnV− extracellular vesicles were higher in patients with a disease duration of less than 10 years compared to those with a disease duration of more than 10 years (91.930 ± 4.183 vs. 85.530 ± 8.343, *p* = 0.0247). Conversely, the CD146− AnnV+ extracellular vesicles were lower in patients with a disease duration of less than 10 years compared to those with a longer disease duration (5.368 ± 4.159 vs. 11.260 ± 8.076, *p* = 0.0145) (Figure 4B).

### 3.3. Effect of Iloprost Infusion on the Percentage of Circulating Extracellular Vesicles

#### 3.3.1. One-Day Infusion

**Figure 5:**
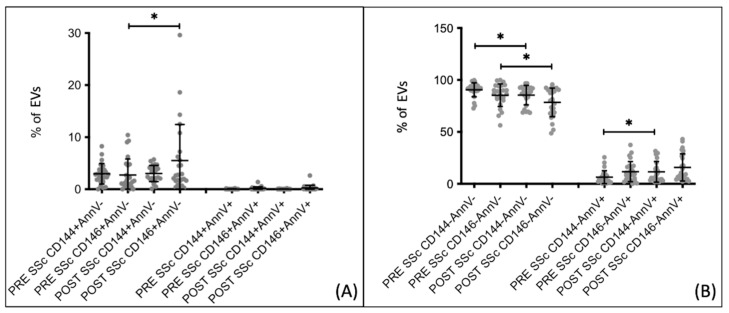
Comparison between percentages of extracellular vesicles before (PRE) and after (POST) Iloprost infusion in SSc patients who follow the 1-day infusion therapeutic scheme. (**A**) eEVs CD146+ from non-apoptotic cells are higher post Iloprost infusion. (**B**) EVs, both CD144− and CD146− from non-apoptotic cells, are lower after infusion, while EVs CD144− from apoptotic cells are higher after infusion. PRE/POST SSc *n* = 25. * *p* < 0.05.

Endothelial extracellular vesicle percentages (CD146+ or CD144+) were compared before and after a 1-day infusion of Iloprost in patients with SSc. A statistically significant difference (*p* = 0.0395) was observed only in CD146+ endothelial extracellular vesicles derived from live/activated cells (AnnV−). The average value of these extracellular vesicles was 2.777 ± 3.071 before the infusion, which then increased to 5.502 ± 6.933 after the drug infusion. However, this difference was not observed when using the CD144 marker, and there were no statistically significant differences in the percentages of apoptotic endothelial extracellular vesicles before and after the infusion (Figure 5A).

Therefore, an 8-h infusion of Iloprost resulted in an increase in extracellular vesicles derived from live/activated endothelial cells.

Regarding non-endothelial extracellular vesicles, those derived from live/activated cells (CD144−/146− AnnV-) decreased after the 1-day Iloprost infusion. The CD144− AnnV− extracellular vesicles decreased from an average of 90.560 ± 6.803 before the infusion to an average of 85.400 ± 9.351 after the infusion (*p* = 0.0172), while the CD146− AnnV− extracellular vesicles decreased from 85.370 ± 10.930 before the infusion to 78.450 ± 13.800 after the infusion (*p* = 0.0496). An increase in CD144− AnnV+ extracellular vesicles, derived from cells undergoing apoptosis, was observed, with an average of 6.446 ± 6.297 before the infusion increasing to 11.560 ± 9.965 after the infusion (*p* = 0.0250) (Figure 5B). However, this increase was not observed for CD146− AnnV+ extracellular vesicles.

**Figure 6:**
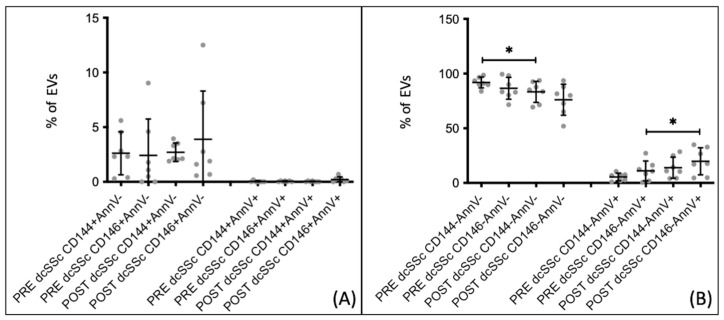
Comparison between percentages of extracellular vesicles before (PRE) and after (POST) Iloprost infusion in dcSSc patients who followed the 1-day infusion therapeutic scheme. (**A**) eEVs, both CD144+ and CD146+, do not show significant differences. (**B**) dcSSc patients show a higher percentage of EVs CD144− from non-apoptotic cells before Iloprost infusion, while EVs CD146− from apoptotic cells are higher after infusion. PRE/POST dcSSc *n* = 7. * *p*-value < 0.05.

Subsequently, extracellular vesicle percentages were analyzed before and after the 1-day Iloprost infusion in different disease subgroups. In patients with dcSSc, no significant changes in the percentages of endothelial extracellular vesicles were observed after the Iloprost infusion (Figure 6A). However, a decrease in CD144− AnnV− extracellular vesicles was noted, with percentages decreasing from 91.880 ± 4.887 before the infusion to 83.330 ± 9.580 after the infusion (*p* = 0.0487). Additionally, there was an increase in CD146− AnnV+ extracellular vesicles, with percentages increasing from 5.476 ± 3.512 before the infusion to 13.930 ± 9.575 after the infusion (*p* = 0.0265) (Figure 6B).

No statistically significant changes in the percentages of extracellular vesicles, both endothelial and non-endothelial, were observed before and after the 1-day Iloprost infusion in patients with the lcSSc.

#### 3.3.2. Five-Day Infusion

When comparing the percentages of extracellular vesicles, both endothelial and non-endothelial, in patients with SSc who received a 5-day infusion of Iloprost, no statistically significant difference was found between the pre- and post-infusion percentages. However, upon analyzing the extracellular vesicles in the two subgroups of the disease, significant changes were observed only in patients with dcSSc.

**Figure 7:**
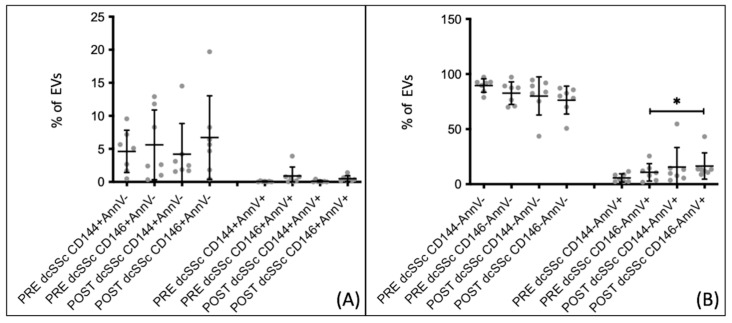
Comparison between percentages of extracellular vesicles before (PRE) and after (POST) Iloprost infusion in dcSSc patients who followed the 5-day infusion therapeutic scheme. (**A**) eEVs, both CD144+ and CD146+, do not show significant differences. (**B**) dcSSc patients show a higher percentage of EVs CD146− from apoptotic cells after infusion. PRE/POST dcSSc *n* = 7. * *p*-value < 0.05.

In patients with dcSSc, there were no changes in endothelial extracellular vesicles following the 5-day Iloprost infusion (Figure 7A). However, non-endothelial apoptotic extracellular vesicles (CD146− AnnV+) exhibited a significant increase (*p* = 0.0482) from 5.727 ± 3.740 before the infusion to 15.600 ± 17.680 after the Iloprost infusion (Figure 7B).

## 4. Discussion

This study investigated the utility of eEVs as potential disease markers in SSc. We found that SSc patients had lower percentages of CD146+ eEVs compared to healthy controls, both for live/activated and apoptotic cells. Additionally, non-endothelial EVs did not exhibit significant differences between SSc patients and controls. 

However, several limitations must be acknowledged. Firstly, the sample size of the study may not adequately represent the larger population of patients with SSc, which is a rare and heterogeneous condition. Secondly, potential biases inherent in the study design, including selection bias and treatment bias, could influence the results due to the varied stages of disease progression and treatment approaches among participants. Nevertheless, it must be considered that the sample size reflects SSc’s status as a rare disease. In addition, SSc is a heterogeneous and chronic disease, which may have various organ involvement over the years requiring different treatments. Our patient cohort, having a long history of the disease, received a range of therapies in addition to Iloprost. This also reflects the clinical reality of a complex and rare disease such as SSc. Additionally, the generalizability of the results is limited by the factors mentioned above and evolving understanding of the role of eEVs in SSc.

The studies conducted on EVs in SSc are not only limited but also report controversial results. The variability in results can be attributed to several factors, such as the utilization of diverse surface markers, e.g., CD144, CD146, CD105, and CD31+/CD42−, for the identification of eEVs and variations in the methodologies applied, with some studies involving the use of frozen samples and others not employing this approach. This heterogeneity makes it challenging to draw direct comparisons and emphasizes the need for standardized approaches in future research to better understand the role of eEVs in SSc pathogenesis [13,32,35,36,37,38,39,40]. The results from this study appear to align with those of other studies [32,35] where the eEVs in SSc patients were quantitatively lower than those among healthy controls. Our study’s findings align with Iversen’s results, which also found decreased eEV levels in SSc using the CD146 marker. Iversen et al. proposed that the decreased levels of eEVs in SSc could be attributed to enhanced extracellular vesicle clearance and their increased adhesion to inflamed endothelial walls. This phenomenon may result in a reduction in EVs freely circulating in the bloodstream in SSc [32]. However, it is important to note that the findings of our study may seem contradictory to some previous research in the field, which reported an increase in eEVs, albeit employing different endothelial markers for identification. Previous studies identified an inverse correlation between skin involvement and eEV levels, suggesting a connection between eEVs and the vascular and fibrotic aspects of SSc. Similar patterns were observed in patients with more severe capillary loss in nailfold capillaroscopy, who exhibited a lower percentage of eEVs [36,37,38]. Endothelial dysfunction and vascular damage are recognized as the initial events in the pathogenesis of SSc, potentially resulting from immune dysregulation and preceding the onset of fibrosis, which is considered an effect of the disease [7]. Different studies support the growing significance of exosomes in regulating the balance between immune tolerance and autoimmunity, emphasizing their involvement in sustaining pathological autoimmune responses. Furthermore, eEVs induced by inflammation have the potential to “educate” target cells, influencing the modulation of growth factor production [41]. In the context of SSc, exosomes released by various immune system components may impact inflammation, a pivotal process in vasculopathy and fibrosis. Elevated levels of exosomes have been observed in SSc patients, and there is speculation about their potential role in modulating endothelial cell apoptosis [11]. Unlike the patients in other studies, most patients enrolled in this study presented advanced disease, far from the early stages characterized by inflammation and edema. The progression of vascular damage in SSc involves the hypertrophy of the tunica intima and media and fibrosis of the adventitia tunica, resulting from the transdifferentiation of endothelial cells and fibroblasts into myofibroblasts, ultimately leading to progressive vessel occlusion [7]. This characteristic process distinguishes SSc from other autoimmune and cardiovascular diseases where EV percentages are higher than in healthy subjects [42]. Circulating endothelial cells and endothelial progenitor cells are elevated in patients with SSc compared to healthy controls as they represent attempts to repair endothelial damage. However, in SSc, these cells exhibit impaired proliferative potential, thus failing to achieve reparative purposes. Consequently, the vessel wall becomes depleted of endothelial cells and populated with fibroblasts and myofibroblasts, leading to progressive vessel occlusion [43]. Considering these mechanisms, the previous studies, and the long history of disease of our patients, we could hypothesize that the eEVs detected in low percentages are the mirror of a long-standing endothelial dysfunction, now evolved into fibrosis. The literature exploring the impact of Iloprost and its mechanisms on EVs is limited. A recent study has shown that prostacyclin analogues play a crucial role in inhibiting platelet reactivity and reducing the release of platelet-derived EVs in patients with PAH. This observation led the authors to hypothesize that modulation of EV concentration may be a key mechanism underlying the therapeutic benefits of prostacyclin analogues in the treatment of PAH. A similar effect was observed with P2Y12 antagonists, which, like prostacyclin analogues, block platelets via a shared intracellular pathway, which involves increasing cAMP levels within cells [44]. One of the key aspects of our study was the evaluation of the impact of Iloprost infusion on eEV percentages. Additionally, there is a lack of data linking the levels of eEVs to infusion therapy with Iloprost, a drug commonly used in this disease to improve the vasculopathic component that primarily affects the microcirculation.

This is the first study that examines eEV percentages before and after Iloprost infusion based on different administration schedules. Following a 1-day infusion, an increase in live-cell-derived eEVs (CD146+, AnnV−) was observed. This uptick supports our previous findings, showing that Iloprost boosts circulating endothelial cells and progenitors as a reparative response to vascular damage [28]. However, after a 5-day infusion, no significant change in eEV percentages was detected. This finding could suggest that the effect of Iloprost on endothelial outcomes might potentially vary with the treatment schedule and that an initial increase in eEVs could be depleted over time. It is worth considering the possible involvement of other factors, such as vesicle clearance mechanisms, which remain poorly understood, that could also contribute to this observation [45]. According to the literature, flow cytometric analysis reveals that the percentage of EVs of endothelial origin constitutes only a small fraction of the total of circulating EVs. On average, approximately 80% of the EVs are derived from platelets, followed by a smaller percentage derived from leukocytes [46]. In our analysis, these subpopulations are represented by EVs negative for the endothelial markers CD144 and/or CD146. They are primarily derived from live/activated cells (AnnV−) and, to a lesser extent, from apoptotic cells (AnnV+). The proportion of eEVs originating from live/activated cells (CD144+/146+ AnnV−) is significantly lower compared to non-endothelial EVs, while apoptotic eEVs (CD144+/146+ AnnV+) are even lower, approaching zero. Our results primarily highlight significant differences in eEVs marked by the CD146 marker rather than those marked by CD144. CD144 is localized solely to the adherent junctions between endothelial cells and is not ubiquitously expressed on the cell membrane [20,22]. Conversely, CD146 is found throughout the membrane, except at adherent junctions where CD144 is localized [21]. The broader distribution of CD146 and its higher expression percentage on endothelial cells make it a more reliable marker for eEVs compared to CD144, which is more specific but less abundantly expressed [47,48].

The variability in findings across studies on EVs in Systemic Sclerosis can be attributed to several factors. These include a lack of standardized flow cytometric methodologies and ongoing debates over the most appropriate endothelial markers to use. Moreover, treatment regimens differ between studies and could affect extracellular vesicle percentage measurement.

The results of this study suggest that eEVs could be markers of endothelial damage in SSc. The observed impact of Iloprost on eEVs, a first in such studies, opens avenues for considering how treatments modulate eEV concentrations and, by extension, disease progression, and symptoms. These insights could lead to more personalized and effective treatment strategies, not only for SSc but potentially for other similar autoimmune and vascular diseases.

## 5. Conclusions

This study suggests that eEVs may reflect the status of the vascular endothelium, appearing as a possible biomarker of vascular damage associated with SSc. This is the first study evaluating the effect of Iloprost infusion on eEV rates. Iloprost administration appears to affect the distribution of eEVs in a way that depends on the specific infusion schedule. Nevertheless, more studies are needed to further investigate the impact of Iloprost on eEVs in SSc and diseases related to vascular endothelium damage. While flow cytometry remains the most employed technique for extracellular vesicle analysis, the absence of a standardized cytofluorimetric protocol hampers the ability to compare extracellular vesicle percentages reported in various scientific studies. Therefore, further research is warranted to establish a well-defined and unanimously accepted reliable cytofluorimetric protocol, allowing for accurate comparisons of extracellular vesicle percentages and, subsequently, defining their utility as potential markers of the disease state. Furthermore, our upcoming objective is to center our attention specifically on vascular dysfunction. Despite the potential introduction of heterogeneity and confounding factors through the inclusion of additional control groups with diverse vasculopathies, this targeted approach aims to furnish more comprehensive insights to ascertain the exclusivity of the observed changes to systemic sclerosis.

## Figures and Tables

**Table 1:** Clinical data of patients included in the study.

SSc patients	*n* = 54
Age (years): mean ± SD	60 ± 13
Sex (F/M)	5.75:1
Disease subset	
Limited cutaneous	33 (61.11%)
Diffuse cutaneous	21 (38.8%)
Autoantibody profile	
ANA positive, *n* (%)	50 (92.5%)
Anti-ENA positive, *n* (%)	22 (40.7%)
Clinical Features	
Pulmonary involvement, *n* (%)	35 (64.8%)
Gastrointestinal involvement, *n* (%)	39 (72%)
Active digital ulcers, *n* (%)	7 (13%)
Secondary Sjogren Syndrome, *n* (%)	24 (44.5%)
Therapy	
Endothelin-receptor antagonists, *n* (%)	12 (22%)
Calcium channel blockers, *n* (%)	28 (52%)
Mycophenolate mofetil, *n* (%)	11 (20%)

## Data Availability

The raw data supporting the conclusions of this article will be made available by the authors, without undue reservation.

## Supplementary Materials

The following supporting information can be downloaded at: https://www.mdpi.com/article/10.3390/biomedicines12020295/s1, Figure S1: Flow cytometric analysis of extracellular vesicles; Figure S2: Comparison between percentages of extracellular vesicles among patients with different capillaroscopic patterns; Figure S3: Comparison between percentages of extracellular vesicles among patients with or without active ulcers; Figure S4: Comparison between percentages of extracellular vesicles among patients with or without pulmonary involvement.

## References

[1] Gabrielli A., Avvedimento E.V., Krieg T. (2009). Scleroderma. N. Engl. J. Med..

[2] Cutolo M., Soldano S., Smith V. (2019). Pathophysiology of Systemic Sclerosis: Current Understanding and New Insights. Expert Rev. Clin. Immunol..

[3] Denton C.P., Khanna D. (2017). Systemic Sclerosis. Lancet.

[4] Argentino G., Barbieri A., Beri R., Bason C., Ruzzenente A., Olivieri O., Tinazzi E., Puccetti A., Vitali C., Del Papa N. (2022). Profibrotic Effects of Endothelin-1 on Fibroblasts Are Mediated by Aldosterone In Vitro: Relevance to the Pathogenesis and Therapy of Systemic Sclerosis and Pulmonary Arterial Hypertension. Biomedicines.

[5] Truchetet M.E., Brembilla N.C., Chizzolini C. (2023). Current Concepts on the Pathogenesis of Systemic Sclerosis. Clin. Rev. Allergy Immunol..

[6] Saketkoo L.A., Distler O. (2012). Is There Evidence for Vasculitis in Systemic Sclerosis. Curr. Rheumatol. Rep..

[7] Mostmans Y., Cutolo M., Giddelo C., Decuman S., Melsens K., Declercq H., Vandecasteele E., De Keyser F., Distler O., Gutermuth J. (2017). The Role of Endothelial Cells in the Vasculopathy of Systemic Sclerosis: A Systematic Review. Autoimmun. Rev..

[8] Cavazzana I., Vojinovic T., Airo’ P., Fredi M., Ceribelli A., Pedretti E., Lazzaroni M.G., Garrafa E., Franceschini F. (2023). Systemic Sclerosis-Specific Antibodies: Novel and Classical Biomarkers. Clin. Rev. Allergy Immunol..

[9] Wermuth P.J., Piera-Velazquez S., Rosenbloom J., Jimenez S.A. (2018). Existing and Novel Biomarkers for Precision Medicine in Systemic Sclerosis. Nat. Rev. Rheumatol..

[10] Giacomelli R., Afeltra A., Alunno A., Bartoloni-Bocci E., Berardicurti O., Bombardieri M., Bortoluzzi A., Caporali R., Caso F., Cervera R. (2019). Guidelines for Biomarkers in Autoimmune Rheumatic Diseases—Evidence Based Analysis. Autoimmun. Rev..

[11] Colletti M., Galardi A., De Santis M., Guidelli G.M., Di Giannatale A., Di Luigi L., Antinozzi C. (2019). Exosomes in Systemic Sclerosis: Messengers between Immune, Vascular and Fibrotic Components?. Int. J. Mol. Sci..

[12] Théry C., Witwer K.W., Aikawa E., Alcaraz M.J., Anderson J.D., Andriantsitohaina R., Antoniou A., Arab T., Archer F., Atkin-Smith G.K. (2018). Minimal Information for Studies of Extracellular Vesicles 2018 (MISEV2018): A Position Statement of the International Society for Extracellular Vesicles and Update of the MISEV2014 Guidelines. J. Extracell. Vesicles.

[13] De Lorenzis E., Rindone A., Di Donato S., Del Galdo F. (2023). Circulating Extracellular Vesicles in the Context of Interstitial Lung Disease Related to Systemic Sclerosis: A Scoping Literature Review. Autoimmun. Rev..

[14] Čolić J., Matucci Cerinic M., Guiducci S., Damjanov N. (2020). Microparticles in Systemic Sclerosis, Targets or Tools to Control Fibrosis: This Is the Question!. J. Scleroderma Relat. Disord..

[15] Leite A.R., Borges-Canha M., Cardoso R., Neves J.S., Castro-Ferreira R., Leite-Moreira A. (2020). Novel Biomarkers for Evaluation of Endothelial Dysfunction. Angiology.

[16] D’anca M., Fenoglio C., Buccellato F.R., Visconte C., Galimberti D., Scarpini E. (2021). Extracellular Vesicles in Multiple Sclerosis: Role in the Pathogenesis and Potential Usefulness as Biomarkers and Therapeutic Tools. Cells.

[17] Mazzucco M., Mannheim W., Shetty S.V., Linden J.R. (2022). CNS Endothelial Derived Extracellular Vesicles Are Biomarkers of Active Disease in Multiple Sclerosis. Fluids Barriers CNS.

[18] Shah R., Patel T., Freedman J.E. (2018). Circulating Extracellular Vesicles in Human Disease. N. Engl. J. Med..

[19] Kalra H., Simpson R.J., Ji H., Aikawa E., Altevogt P., Askenase P., Bond V.C., Borras F.E., Breakefield X., Budnik V. (2012). Vesiclepedia: A Compendium for Extracellular Vesicles with Continuous Community Annotation. PLoS Biol..

[20] Giannotta M., Trani M., Dejana E. (2013). VE-Cadherin and Endothelial Adherens Junctions: Active Guardians of Vascular Integrity. Dev. Cell.

[21] Bardin N., Anfosso F., Massé J.M., Cramer E., Sabatier F., Le Bivic A., Sampol J., Dignat-George F. (2001). Identification of CD146 as a Component of the Endothelial Junction Involved in the Control of Cell-Cell Cohesion. Blood.

[22] Gerhardt T., Ley K. (2015). Monocyte Trafficking across the Vessel Wall. Cardiovasc. Res..

[23] Venable A.S., Williams R.R., Haviland D.L., Mcfarlin B.K. (2014). An Analysis of Endothelial Microparticles as a Function of Cell Surface Antibodies and Centrifugation Techniques. J. Immunol. Methods.

[24] Wang Z., Yan X. (2013). CD146, a Multi-Functional Molecule beyond Adhesion. Cancer Lett..

[25] Wlodkowic D., Skommer J., Darzynkiewicz Z. (2012). Cytometry of Apoptosis. Historical Perspective and New Advances. Exp. Oncol..

[26] Dunne J., Bankole J., Keen K. (2014). Systematic Review of the Role of Microparticles in Systemic Sclerosis. Curr. Rheumatol. Rev..

[27] Kowal-Bielecka O., Fransen J., Avouac J., Becker M., Kulak A., Allanore Y., Distler O., Clements P., Cutolo M., Czirjak L. (2017). Update of EULAR Recommendations for the Treatment of Systemic Sclerosis. Ann. Rheum. Dis..

[28] Tinazzi E., Dolcino M., Puccetti A., Rigo A., Beri R., Valenti M., Corrocher R., Lunardi C. (2010). Gene Expression Profiling in Circulating Endothelial Cells from Systemic Sclerosis Patients Shows an Altered Control of Apoptosis and Angiogenesis That Is Modified by Iloprost Infusion. Arthritis Res. Ther..

[29] Van Den Hoogen F., Khanna D., Fransen J., Johnson S.R., Baron M., Tyndall A., Matucci-Cerinic M., Naden R.P., Medsger T.A., Carreira P.E. (2013). 2013 Classification Criteria for Systemic Sclerosis: An American College of Rheumatology/European League against Rheumatism Collaborative Initiative. Arthritis Rheum..

[30] LeRoy E.C., Black C., Fleischmajer R., Jablonska S., Krieg T., Medsger T.A., Rowell N., Wollheim F. (1988). Scleroderma (Systemic Sclerosis): Classification, Subsets and Pathogenesis. J. Rheumatol..

[31] LeRoy E.C., Medsger T.A. (2001). Criteria for the Classification of Early Systemic Sclerosis. J. Rheumatol..

[32] Iversen L.V., Ostergaard O., Ullman S., Nielsen C.T., Halberg P., Karlsmark T., Heegaard N.H.H., Jacobsen S. (2013). Circulating Microparticles and Plasma Levels of Soluble E- and P-Selectins in Patients with Systemic Sclerosis. Scand. J. Rheumatol..

[33] Rodríguez-Carrio J., Alperi-López M., López P., Alonso-Castro S., Carro-Esteban S.R., Ballina-García F.J., Suárez A. (2015). Altered Profile of Circulating Microparticles in Rheumatoid Arthritis Patients. Clin. Sci..

[34] Sellam J., Proulle V., Jüngel A., Ittah M., Miceli Richard C., Gottenberg J.-E., Toti F., Benessiano J., Gay S., Freyssinet J.-M. (2009). Increased Levels of Circulating Microparticles in Primary Sjögren’s Syndrome, Systemic Lupus Erythematosus and Rheumatoid Arthritis and Relation with Disease Activity. Arthritis Res. Ther..

[35] Jung C., Drummer K., Oelzner P., Figulla H.R., Boettcher J., Franz M., Betge S., Foerster M., Wolf G., Pfeil A. (2016). The Association between Endothelial Microparticles and Inflammation in Patients with Systemic Sclerosis and Raynaud’s Phenomenon as Detected by Functional Imaging. Clin. Hemorheol. Microcirc..

[36] De Oliveira S.M., Luiz I., Teixeira D.A., França C.N., Cristina M., Izar D.O., Kayser C. (2023). Microparticles: Potential New Contributors to the Pathogenesis of Systemic Sclerosis?. Adv. Rheumatol..

[37] Guiducci S., Distler J.H.W., Jüngel A., Huscher D., Huber L.C., Michel B.A., Gay R.E., Pisetsky D.S., Gay S., Matucci-Cerinic M. (2008). The Relationship between Plasma Microparticles and Disease Manifestations in Patients with Systemic Sclerosis. Arthritis Rheum..

[38] Michalska-Jakubus M., Kowal-Bielecka O., Smith V., Cutolo M., Krasowska D. (2017). Plasma Endothelial Microparticles Reflect the Extent of Capillaroscopic Alterations and Correlate with the Severity of Skin Involvement in Systemic Sclerosis. Microvasc. Res..

[39] Jud P., Meinitzer A., Strohmaier H., Schwantzer G., Foris V., Kovacs G., Avian A., Odler B., Brodmann M., Hafner F. (2021). Evaluation of Endothelial Dysfunction and Clinical Events in Patients with Early-Stage Vasculopathy in Limited Systemic Sclerosis. Clin. Exp. Rheumatol..

[40] Leleu D., Levionnois E., Laurent P., Lazaro E., Richez C., Duffau P., Blanco P., Sisirak V. (2020). Elevated Circulatory Levels of Microparticles Are Associated to Lung Fibrosis and Vasculopathy During Systemic Sclerosis. Front. Immunol..

[41] Yamamoto S., Niida S., Azuma E., Yanagibashi T., Muramatsu M., Huang T.T., Sagara H., Higaki S., Ikutani M., Nagai Y. (2015). Inflammation-Induced Endothelial Cell-Derived Extracellular Vesicles Modulate the Cellular Status of Pericytes. Sci. Rep..

[42] Šibíková M., Živný J., Janota J. (2018). Cell Membrane-Derived Microvesicles in Systemic Inflammatory Response. Folia Biol..

[43] Del Papa N., Pignataro F. (2018). The Role of Endothelial Progenitors in the Repair of Vascular Damage in Systemic Sclerosis. Front. Immunol..

[44] Gąsecka A., Banaszkiewicz M., Nieuwland R., van der Pol E., Hajji N., Mutwil H., Rogula S., Rutkowska W., Pluta K., Eyileten C. (2021). Prostacyclin Analogues Inhibit Platelet Reactivity, Extracellular Vesicle Release and Thrombus Formation in Patients with Pulmonary Arterial Hypertension. J. Clin. Med..

[45] Ayers L., Nieuwland R., Kohler M., Kraenkel N., Ferry B., Leeson P. (2015). Dynamic Microvesicle Release and Clearance within the Cardiovascular System: Triggers and Mechanisms. Clin. Sci..

[46] Flaumenhaft R., Dilks J.R., Richardson J., Alden E., Patel-Hett S.R., Battinelli E., Klement G.L., Sola-Visner M., Italiano J.E. (2009). Megakaryocyte-Derived Microparticles: Direct Visualization and Distinction from Platelet-Derived Microparticles. Blood.

[47] Dignat-George F., Sabatier F., Blann A., Woywodt A. (2007). Detection of Circulating Endothelial Cells: CD146−Based Magnetic Separation Enrichment or Flow Cytometric Assay?. J. Clin. Oncol..

[48] Jacques N., Vimond N., Conforti R., Griscelli F., Lecluse Y., Laplanche A., Malka D., Vielh P., Farace F. (2008). Quantification of Circulating Mature Endothelial Cells Using a Whole Blood Four-Color Flow Cytometric Assay. J. Immunol. Methods.

