# Supplementary material for: Exploring the Utility of Circulating Endothelial Cell-Derived Extracellular Vesicles as Markers of Health and Damage of Vasal Endothelium in Systemic Sclerosis Patients Treated with Iloprost
Source: Biomedicines. 2024 Jan 27;12(2):295. doi: 10.3390/biomedicines12020295 (PMC10886571; doi:10.3390/biomedicines12020295)
Supplement: Supplementary file 1 [file biomedicines-12-00295-s001.zip › biomedicines-2835437-supplementary.pdf]

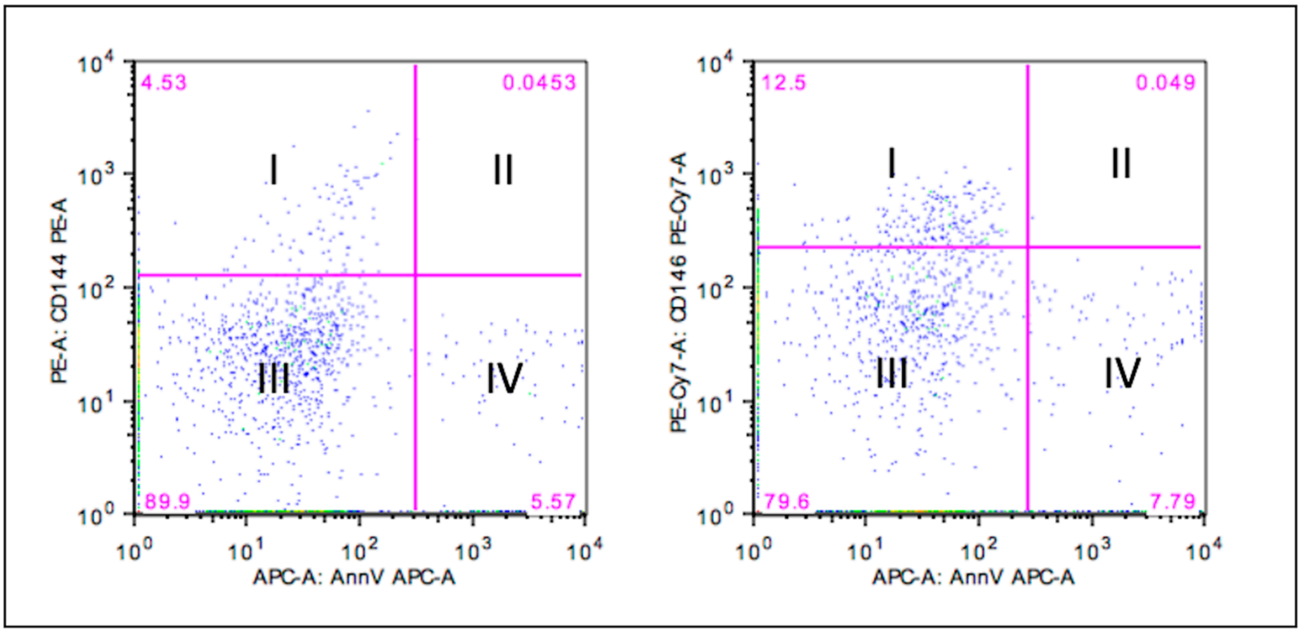

Supplementary Figure S1: Flow cytometric analysis of extracellular vesicles. The extracellular vesicles included in the gate were further subdivided based on the expression of CD144 or CD146 and AnnV on their surface: I) extracellular vesicles derived from normal endothelial cells (CD144+/146+ AnnV-), II) extracellular vesicles derived from apoptotic endothelial cells (CD144+/146+ AnnV+), III) extracellular vesicles derived from non-endothelial cells, both viable (CD144-/146- AnnV-) and IV) apoptotic (CD144-/146- AnnV+)

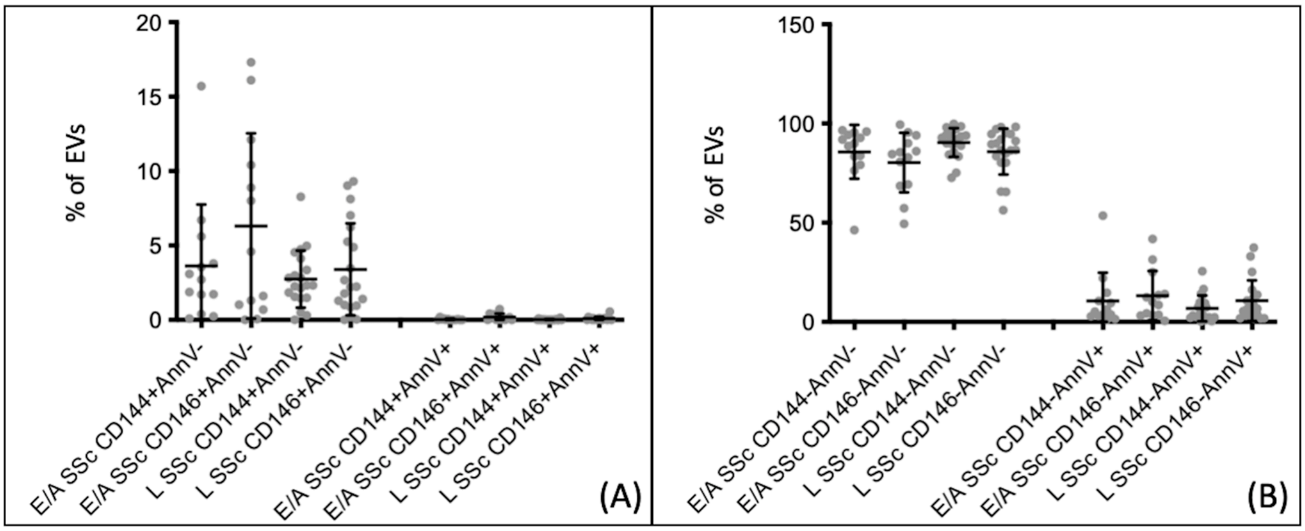

Supplementary Figure S2: Comparison between percentages of extracellular vesicles among patients with different capillaroscopic patterns. Patients were divided into 2 patterns: “early/active” (E/A) and "late" (L). No significant differences are found both in eEVs (A) and in EVs (B). E/A SSc n=13, L SSc n=20.

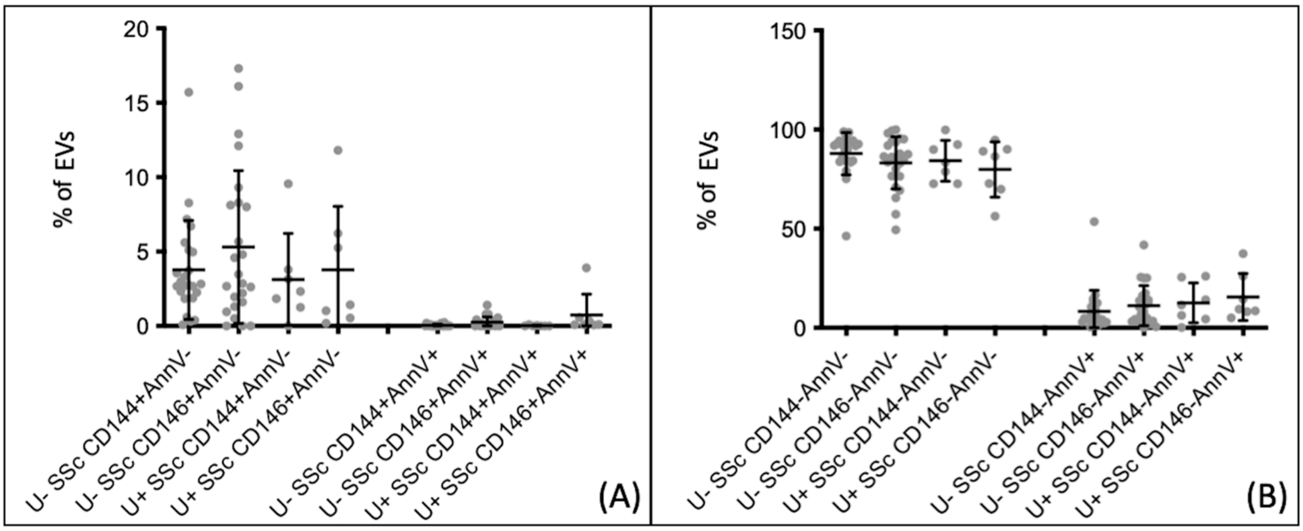

Supplementary Figure S3: Comparison between percentages of extracellular vesicles among patients with or without active ulcers. Patients were divided into 2 groups: "absence of active ulcerous" (U-) and "presence of active ulcerous" (U+). No significant differences are found both in eEVs (A) and in EVs (B). U- SSc=24, U+ SSc n=7.

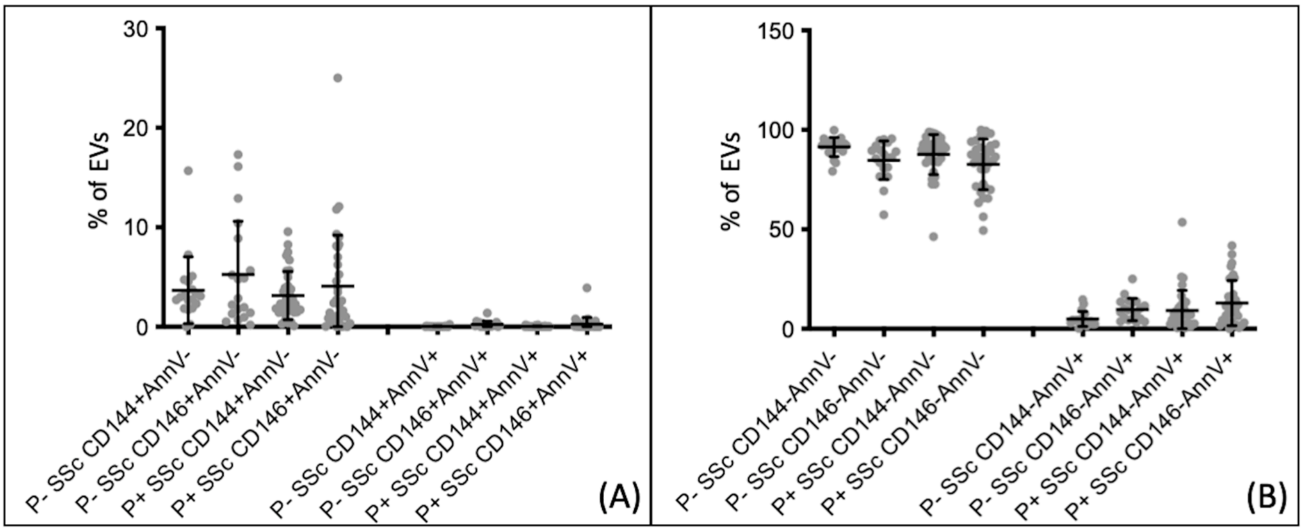

Supplementary Figure S4: Comparison between percentages of extracellular vesicles among patients with or without pulmonary involvement. Patients were divided into 2 groups: "absence of pulmonary involvement" (P-) and "presence of pulmonary involvement" (P+). No significant differences are found both in eEVs (A) and in EVs (B). P- SSc n=19, P+ SSc n=35.
